# Chemosensory Systems in COVID-19: Evolution of Scientific Research

**DOI:** 10.1021/acschemneuro.0c00788

**Published:** 2021-02-09

**Authors:** Sheila Veronese, Andrea Sbarbati

**Affiliations:** Department of Neuroscience, Biomedicine and Movement Sciences, University of Verona, 37134 Verona, Italy

**Keywords:** Anosmia, chemosensory disfunctions, chemosensory systems, disease severity, neurodegeneration, neurocovid

## Abstract

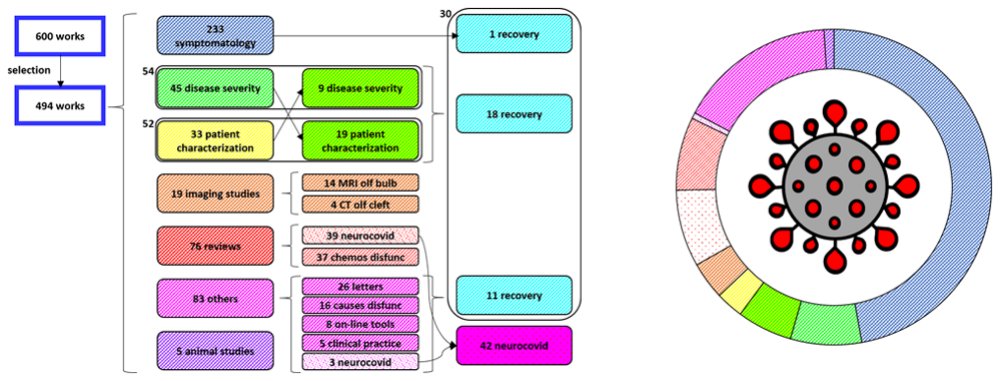

COVID-19 disease induced by coronavirus SARS-CoV-2 presents among its symptoms alterations of the chemosensory functions. In the first studies on the Chinese population, this symptomatology was not particularly relevant, and hyposmia and hypogeusia were excluded from the symptoms to be evaluated to diagnose the disease. With the pandemic spread of the illness, there has been an augment in reports on chemosensory dysfunctions among patients. The first data analysis showed the presence of these disorders mainly in paucisymptomatic and asymptomatic patients. The interest in chemosensory systems therefore increased considerably, because the olfactory and gustatory symptoms could be the key to stop the infection spread. Furthermore, the degree of alert and attention grew, considering that these types of dysfunctions are prognostic symptoms of serious neurodegenerative diseases. About 9 months have passed since the first anecdotal reports on the involvement of the olfactory and gustatory systems in the COVID-19 pathology. For this reason, a careful review of the literature was conducted to understand if it is clearer which people present chemosensory symptoms and if these are related to the severity of the disease. Furthermore, we have identified which aspects still remain to be clarified.

A new coronavirus has spread through the human population since December 2019. This new virus was classified as severe acute respiratory syndrome coronavirus-related 2 (SARS-CoV-2) by the Coronaviridae Study Group of the International Committee on Taxonomy of Viruses.^1^ The disease caused by SARS-CoV-2 was named “Coronavirus disease 19” (COVID-19) by the World Health Organization (WHO) on February 11, 2020. From the area of origin, the city of Wuhan, in the Hubei province in China,^2,3^ COVID-19 has spread all over the world in a particularly rapid and aggressive way. Due to the high contagion and lethality rates, the WHO declared the world pandemic state on March 11, 2020.

In the first studies on the Chinese population, there was no evidence of patients with symptoms related to alterations and/or loss of smell and taste^4−6^ or of the percentages of the subjects where hyposmia and hypogeusia were excluded from the symptoms to be evaluated to diagnose the disease.^7^ This may be attributable both to the criticality of the general picture of the first patients, presenting serious respiratory symptoms, often associated with important comorbidities, and to the general difficulty in identifying smell and taste disorders.^8−10^

With the spread of the virus outside China and the rapid increase in COVID-19 cases, there has been an increase in reports of olfactory and gustatory symptoms, first in anecdotal form, then with case reports, and finally with scientific studies. The increase in reported cases of hyposmia, anosmia, and ageusia could be linked to attention being directed to this symptomatology, with consequent more accurate research of these symptoms among infected patients. This increase could also reflect a real increase in the incidence, related to genetic mutations found in SARS-CoV-2 in the same Chinese territory and during the spread from China to the rest of the world.^11,12^

From the first data relating to the olfactory and gustatory symptoms, the fact that they seemed more present in asymptomatic or paucisymptomatic subjects appeared of particular interest.^13−15^ In fact, these types of symptoms could be the key to identifying asymptomatic carriers and reducing the number of new infections, favoring the containment of the spread of SARS-CoV-2. This possibility, combined with the growing number of patients reporting this symptomatology, led to the insertion, in particular, of anosmia among the symptoms of COVID-19, primarily by the British Rhinological Society and the ENT UK organization^16^ and by the American Academy of Otolaryngology - Head and Neck Surgery (AAO-HNS).^17^ The ENT UK organization warned all individuals with anosmia and no other symptoms to proceed with precautionary self-isolation. The AAO-HNS specified that anosmia, hyposmia, and dysgeusia in the absence of other respiratory diseases such as allergic rhinitis, acute rhinosinusitis, or chronic rhinosinusitis should advise doctors of the possibility of COVID-19 infection and justify a serious consideration for the self-isolation and testing of individuals with these symptoms.

Anosmia was the subject of discussion for a variety of reasons, from the determination of the time (number of days) and the degree (total or partial) of recovery of the olfactory functionality,^13,15^ to the real correlation with COVID-19, given the known existence of a wide range of viral infections that cause an inflammatory-type immune reaction and a response of the nasal mucosa with consequent development of rhinorrhea and olfactory dysfunctions.^18−20^

Furthermore, the degree of alert and attention increased, considering that olfactory dysfunctions are prognostic symptoms of serious neurodegenerative diseases, such as Parkinson’s^21−24^ and Alzheimer’s diseases^25,26^ but also Creutzfeldt-Jakob disease^27^ and neuroimmunological diseases such as multiple sclerosis, optic neuromyelitis, and systemic lupus erythematosus.^28^ The spectrum of the neuroinvasive potential of SARS-CoV-2, of possible immediate neural damage, but also of short- and long-term neurological sequelae became real.^29,30^

About 9 months have passed since the first anecdotal reports on the involvement of the olfactory and gustatory systems in the COVID-19 pathology. For this reason, a careful review of the literature is useful to understand if the above questions have been answered, if it is clearer which people have chemosensory symptoms, if these are related to the severity of the disease, and which aspects still remain to be clarified.

## First Evidence of Chemosensory Systems Involvement in COVID-19

As of February 2020, there have been anecdotal reports of smell and taste loss associated with COVID-19 around the world, but formal scientific studies began in March.

One of the first research studies was conducted in Iran, one of the first countries affected by SARS-CoV-2 and where the wave of olfactory dysfunctions in patients referred to ENT clinics was noted along with the increase in the number of COVID-19 cases. Between March 12 and 17, 2020, through a cross-sectional analysis with an online checklist on voluntary cases in all provinces of Iran, more than 10 000 subjects were evaluated and a significant correlation was identified between anosmia and COVID-19 positivity.^31^

## March 20, 2020

On March 20, 2020, the formal position taken by ENT UK^16^ and, 2 days later, by the AAO-HNS^17^ on the relevance of chemosensory symptoms explicated the correlation with COVID-19, even in the absence of other symptoms, and allowed the launch of targeted studies. The echo of these statements was reflected in the main scientific journals and the hypothesis of the neuroinvasiveness of SARS-CoV-2 through the olfactory bulb was immediately formulated.^30,32^

Some authors began to report observational data on symptoms of loss of taste and smell in COVID-19 patients, recommending caution for health workesr and particular attention in evaluating this symptomatology in all patients and especially in the most fragile ones,^33^ considering the evidence that it could be the only symptom present or concomitant to mild other signs.^34,35^ However, in the first phase of alert against chemosensory symptoms, some authors recommended prudence in their association with COVID-19,^36^ also to avoid forms of self-isolation without appropriate reports to the competent doctors.^37^

The British group headed by Prof. Hopkins tried to link these symptoms to COVID-19 using both epidemiological analysis^38^ and Google trends^39^ in order to associate the increase in the incidence of olfactory and gustatory symptoms in the British population to the increase in COVID-19 cases. The ability to detect early mild symptoms of the disease was obviously desirable to prevent more severe and lethal forms of COVID-19. On the other hand, identifying the presence of these symptoms in asymptomatic or paucisymptomatic patients was fundamental to avoid a wider spread of the infection. These observations prompted the first reviews of the literature aimed at finding articles focused on sinonasal pathophysiology in COVID-19^40^ and underlining the importance of a more scientific focus on these symptoms, compared to social media.^41^

## Patient Reports of Symptoms: First Month Data

The first study on a group of 59 hospitalized patients was conducted in Milan, Italy, one of the countries most heavily affected by the virus, and is dated March 26, 2020.^42^ In this study, 33.9% of these patients reported at least one taste or olfactory disorder and 18.6% reported both; 60% of symptomatic patients had manifestations before hospitalization. The symptoms were more present in women and young patients.

The articles published subsequently were single^43−45^ or few^46−49^ case reports or studies on patient groups. Regarding the single or few case reports, the data of the various patients are shown in Table 1.

**Table 1:** Chemosensory Symptomatology of Patients from Single and Few Case Reports^a^

		partial			total			
age	gender	hypogeusia	hyposmia	both	ageusia	anosmia	both	other symptoms
<45 y	F			X				myalgias
	F			X				myalgias
	M			X				
	M			X				
	F		X					cough, fever
	F					X		abdominal pain, chills, cough, diarrhea, headache, myalgias
	F					X		cough, fever
	F					X		cough, nasal obstruction, runny nose, severe headache, sore throat
	M						X	dry cough, fever, mucus, muscle aches, slight nasal obstruction
45–65 y	F					X		
	F						X	
	M						X	
	F			X				allergic rhinitis
	F					X		cephalagia, cough, myalgias
>65 y	M	dysgeusia						respiratory symptoms
	M					X		fatigue
	F		X		X			fatigue

a F, female; M, male; y, years.

A total of 17 COVID-19 patients were presented. There was a prevalence of females (65%) and patients <45 years of age (53%), while patients> 65 years of age represented 18% of the population. Seven subjects presented partial dysfunctions, 9 showed total dysfunctions, and one patient showed ageusia and hyposmia. And 94% of patients presented smell disorders, while only the 59% had taste dysfunctions. Only a patient presented dysgeusia and no olfactory symptoms. In addition, 71% of the subjects presented other symptomatology, that is, 29% had only chemosensorial manifestations.

The data of studies in larger patient groups are reported in Table 2. A total of 1040 COVID-19 patients were involved, of which 476 (45.8%) were tested online through questionnaires. The disease appears to be predominantly mild to moderate. Five out of eight studies reported comorbidities of the respiratory type. In all studies, fever and cough were detected as symptoms being present. Six out of eight studies reported myalgia and fatigue problems (fatigue, shortness of breath, and weakness), while five out of eight studies reported headaches. Unlike single or few case reports, there was no noticeable numerical discrepancy between patient data reporting olfactory and gustatory dysfunctions. And 50% of the studies reported the recovery time of chemosensory dysfunctions, generally from the resolution of COVID-19 symptoms and, mainly, within 2 weeks; 3–4 weeks was also reported for total recovery, but not all patients had a complete restoration of function (neither partial nor total).

**Table 2:** Etiological Data of Patients Included in Large Group Studies^a^

study	patients	type	% other symptoms (first five listed)	% comorbidity (>5% listed)	% incidence	duration chemosensory symptoms	recovery time chemosensory disorders
Gelardi et al.^50^	72	patients visit in primary care setting	86 fever; 80 cough; 47 breathlessness; 40 weakness; 22 headache	NA	only anosmia 11; Only dysgeusia 18; both anosmia and dysgeusia 47	16.1 days (range 7–22)	resolution in 49% of patients in 22 days (range 8–29)
Klopfenstein et al.^51^	114	37% hospitalized; 9% hospitalized in intensive care unit; 20% oxygen therapy; 4% death	93 fatigue; 87 cough; 82 headache; 74 fever; 74 myalgia	13 hypertension; 13 asthma; 11 cardiovascular disease; 6 chronic obstructive pulmonary disease	dysgeusia 85; anosmia 47	NA	16%, 1–3 days; 30%, 4–6 days; 35%, 7–13 days; 14%, 14–20 days; 5%, 21–27 days; 1% no recovery
Lechien et al.^13^^b^	417	mild to moderate	>75 cough;^c^ >55 myalgia;^c^ >50 anorexia;^c^ >50 diarrhea;^c^ >45 fever^c^	15 allergic rhinitis;^c^ 7.5 asthma;^c^ 6 hypertension^c^	anosmia 68.1; hyposmia 17.5; hypogeusia 64.7; dysgeusia 17.3; does not remember smell symptom 7.7; only anosmia 3.8; only hyposmia 1.0	NA	*olfactory disorders*: 33%, 1–4 days; 39.6%, 5–8 days; 24.2%, 9–14 days; 3.3%, >15 days
							*only anosmia*: 20.3%, 1–4 days; 47.5%, 5–8 days; 28.8%, 9–14 days; 3.4%, >15 days
Levinson et al.^52^	42	mild	69 cough; 69 fatigue; 66.6 fever 57 myalgia; 21.4 diarrhea	9.5 did not specify	anosmia 35.7; dysgeusia 33.3; anosmia and dysgeusia 33.3; only anosmia 2.4	dysgeusia 7.1 days (range 0–7); anosmia 7.6 days	NA
Mao et al.^14^^d^	214	58.9% nonsevere; 41.1% severe	61.7 fever; 50.0 cough; 31.8 anorexia; 19.2 diarrhea; 14.5 throat pain	23.8 hypertension; 14.0 diabetes; 7.0 cardiac or cerebrovascular disease; 6.1 malignancy	taste 5.6 (3.4 severe, 7.1 non severe); smell 5.1 (3.4 severe, 6.3 non severe)	NA	NA
Moein et al.^53^	60	10% severe; 48% moderate; 42% mild	46.8 fever; 35.6 cough; 31.5 breathlessness; 22.4 headache; 5.8 myalgia	13.3 diabetes; 10 hypertension; 6.7 autoimmune; disease; 6.7 hypothyroidism; 5 asthma	anosmia 25; hyposmia 73; dysfunction of both taste and smell 35; only smell 12; only taste 7; loss of both taste and smell 17	NA	NA
Yan et al.^15^^b^	59	6.9% hospitalized	81.4 fatigue; 69.5 fever; 66.1 cough; 66.1 headache; 62.7 myalgia	33.9 allergic rhinitis; 15.3 immunosuppressed state; 13.6 hypertension; 8.5 diabetes; 5.1 cardiac disease; 5.1 chronic lung disease	ageusia 71.2; anosmia 67.8	NA	Smell: 72% (only 40 patients) improvement at the time of survey (18%, <1 week; 37.5%, 1–2 weeks; 18%, 2–4 weeks)
Zayet et al.^54^	62	47% hospitalized	94 fatigue; 81 cough; 78 headache; 76 fever; 61 myalgia	35 cardiovascular disease; 18 obstructive pulmonary disease; 16 diabetes	anosmia 52; dysgeusia 48	NA	NA

a NA, not available.

b Online questionnaire.

c Data obtained from graphs.

d Data retrospectively collected from medical files - possible underestimation of real prevalence.

The data showed something interesting: the onset time of the chemosensory signs. Giacomelli et al.^42^ reported that 60% of patients presented the symptoms before hospital admission, whereas 40% experienced the symptoms during their hospital stay. In the group of studies of Table 2, Gelardi et al.^50^ reported onset before respiratory manifestations, while other authors referred concurrent onset with other symptoms,^13,52,53^ and Klopfenstein et al.^51^ reported onset after the other signs and during the disease. The difference in these data can be linked to the difficulty in identifying and testing this type of symptomatology. However, this difficulty is generally present, and not only for patients with COVID-19, and it is mostly present during the acute phases of diseases.^55^

Finally, we detected two works relating the retrospective evaluation of data collected from patients via a web-based questionnaire. Both studies found a strong association between chemosensory symptomatology and COVID-19.^56,57^

## Olfactory Route for Virus Entry into Central Nervous System: First Month Data

The invasion of the central nervous system (CNS) along the olfactory pathway had been proven for Middle East Respiratory Syndrome-related Coronavirus (MERS-CoV) and Severe Acute Respiratory Syndrome-related Coronavirus (SARS-CoV), both belonging to the coronavirus family, both in animals^32,58^ and in humans.^59,60^ For this reason, a series of studies tried to explain the different possible mechanisms of SARS-CoV-2 invasion of the CNS, including the olfactory pathway, and to predict the possible central damages.

Table 3 summarizes the main contents and conclusions of the works in which the authors speculated on the possible access modalities of SARS-CoV-2 in the CNS. The olfactory nerve is one of the possible ways of accessing the CNS, but it is not the only one. The authors reported the retrograde neural route and the trans-synaptic pathway. Furthermore, from non-neural olfactory epithelium cells, the virus may pass directly to cerebrospinal fluid near the cribriform plate close to the olfactory bulb. Moreover, lymphatic and blood pathways are possible. For the second one, the virus would spread through the blood via crossing endothelial cells that express the angiotensin converting enzyme 2 (ACE2) receptor, the SARS-CoV-2 receptor. The common conclusion of all the works in Table 3 was that SARS-CoV-2 could cause neurologic complications. The eventual neural damages could result in dysfunction, such as olfactory and gustatory, but also aggravate the morbidity and mortality caused by COVID-19.

**Table 3:** Access Modalities of SARS-CoV-2 in the CNS^a^

study	main contents	main conclusions
Aaroe et al.^61^	Neurological manifestations in COVID-19 are common to symptoms of other CoVs, for which the invasion of the CNS has been highlighted. Among the routes of entry to the CNS, they remember the hematogenous and neural route and the olfactory bulb.	For neuro-oncologists, COVID-19 represents an increased mortality risk in cancer patients.
Baig et al.^62^	ACE2, the SARS-CoV-2 receptor, has been identified on glial cells and neurons in the human brain. SARS-CoV-2 neurotropism can occur through circulation and/or an upper nasal transcribrial pathway that allows COVID-19 to reach the brain and to bind and engage with ACE2 receptors.	There is a possible contribution of neurological tissue damage to olfactory and gustatory disfunctions and in the morbidity and mortality caused by COVID-19.
Butowt and Bilinska^29^	The olfactory epithelium may be a site of SARS-CoV-2 replication, accumulation, and brain entrance through an anterograde axonal transport along the olfactory nerve. Harberts et al.^63^ are cited to suggest an alternative brain invasion route: from non-neural olfactory epithelium cells, the virus may pass directly to cerebrospinal fluid near the cribriform plate and reach most of the brain areas including the medulla oblongata where cardiorespiratory controlling nuclei are located.	Olfactory dysfunctions can result from invasion of the olfactory epithelium of SARS-CoV-2. Brain infections can begin in olfactory neurons.
Cevik et al.^64^	To summarize the different possible access route to the brain, they cited Mao et al.^14^ (neuronal involvement of areas in proximity to the olfactory bulb), Li et al.^30^ (trans-synaptic transfer in the usage of neuroanatomic interconnections of the respiratory and gastrointestinal system to the nuclei of the brainstem), and Baig et al.^62^ (dissemination through the blood via crossing endothelial cells that express the ACE2 receptor).	There is growing evidence that the brain could be the main trigger in the severity of COVID-19.
Conde et al.^65^	It is emphasized that the olfactory pathway may be the possible way of entry of SARS-CoV-2 into the brain. Some clinical evidence of brain impairment is reported in the literature,^66−68^ and the case of a patient with massive intracerebral bleeding is presented.	Respiratory distress is not only the result of pulmonary inflammatory structural damage, but also a result of the damage caused by the virus in the respiratory centers of the brain.
Eliezer et al.^43^	A case of anosmia without nasal obstruction is described. They cited Li et al.^30^ to speculate on the infection mechanisms for SARS-CoV-2 via the cribriform plate close to the olfactory bulb and the olfactory epithelium, and Yao et al.^69^ to suggest MRI evaluation of olfactory bulb volume as a reduction is inversely related to the duration of olfactory loss.	SARS-CoV- 2 may infect the brain, and anosmia, without nasal obstruction with other symptoms, can be an indication of infection.
Li et al.^30^	CoVs can spread via a synapse-connected route to the medullary cardiorespiratory centers in the brainstem. A dysfunction of these centers may cause the death of infected animals and patients.	CoVs have a neuroinvasive propensity. Neurologic manifestations in COVID-19 indicate that SARS-CoV-2 could induce respiratory failure.
Machado and Gutierrez^70^	Revision of the possible mechanisms of smell and taste loss in COVID-19 with the hypothesis that taste loss may be secondary and derived from olfactory loss. They suggest the possibility of CNS invasion through the olfactory route.	People who experience smell and/or taste loss, even as unique symptoms, should be considered as potential SARS-CoV-2 carriers.
Mao et al.^14^	Symptomatology of 214 patients is present. The expression and distribution of ACE2 may cause some neurological manifestations. As with other respiratory viruses, SARS-CoV-2 may enter the CNS through the hematogenous or retrograde neural route.	SARS-CoV-2 presents central and peripheral nervous system manifestations.
Wu et al.^71^	CoVs have been detected in the cerebrospinal fluid and in the brain. CoVs can enter the nervous system through the olfactory nerve, the blood circulation, and neuronal pathways. Thus, CoVs can cause nerve damage through direct infection pathways, but also hypoxia, immune injury, ACE2, and other mechanisms.	SARS-CoV-2 may cause neurological diseases.
Zegarra-Valdivia et al.^72^	Review of the literature on possible access routes to the CNS. They cited Desforges et al.,^59,60^ Li et al.^30^ and Baig et al.^62^ to summarize the trans-synaptic pathway, the blood and lymphatic pathways, the diffusion through the cribriform plate. CoVs can exacerbate neurological symptoms and cause neurodegeneration and death.	COVID-19 may have neurological complications that may last longer than the infection itself.

a ACE2, angiotensin-converting enzyme 2; CNS, central nervous system; CoV, coronavirus; MRI, magnetic resonance images.

It should be noted that a different hypothesis on the method of invasion of the CNS and of the possible multiorgan dysfunctions induced by SARS-CoV-2 was described by Wickramaratchi et al.,^73^ which indicated the pancreas as the first target organ of the virus and the propagation along the afferent part of the vagus nerve (which controls the pancreatic enzyme secretions) for central access. After the central invasion, chemosensory alteration and virus propagation to other organs were hypothesized. The latter implied the manifestation of different conditions, such as upper respiratory tract infection.

## First Neural Complication Hypothesis: Should We Talk about NeuroCovid?

Having identified all possible ways to access the CNS, the possibility of having neurological complications was argued, in another scientific line of publications consisting of five editorials/opinions and two papers on ACE2. The five comments, given the consistency of the first signs of neural invasiveness of SARS-CoV-2, highlighted the possible aggravation of the future neurological picture of patients with COVID-19 and underlined the importance of the work that the neurologists will have at the end of the pandemic.^74−78^

The other two articles came to the same conclusions by analyzing the distribution of ACE2 in animal and human brains. In mice, ACE2 is expressed in neurons of the paraventricular nucleus, in the postrema area, in the dorsal motor nucleus of the vagus, in the solitary tractus nucleus, in the rostroventrolateral marrow, and in the ambiguous nucleus, all brain structures related to cardiovascular and respiratory function.^79^ The authors stated that neurobiological sequelae, which can induce alterations in these areas, could cause the transformation of COVID-19 into a real NeuroCoViD-19.^80,81^

## The Neuroinvasion: First Evidence

At this point, following the observation of the possible neuroinvasiveness of SARS-CoV-2 and given the neurological symptoms, both central and peripheral, in COVID-19, scientists went in search of possible neural damage in patients. Consequently, a whole series of articles, including damage confirmations even on single patients, was published.

Analysis of the first autopsy data from Chinese patients with COVID-19 revealed that brain tissue was hyperaemic and oedematous and that some neurons had degenerated.^14^ The cases of living patients are reported in Table 4. Ten cases are reported. There was a prevalence of male cases with five male and two female subjects, in the six studies in which the sex of the patients was specified. Patient age was detailed in eight studies, for nine subjects: two patients were >45 years old, four were aged between 45 and 65 years, and three others were >65 years old.

**Table 4:** Neural Damage in Patients Affected by COVID-19^a^

study	case	age	gender	chemosensory symptoms	imaging findings
Conde et al.^65^		79			massive intracerebral bleeding from the right hemisphere
Filatov et al.^66^	1 encephalopathy	74			no acute abnormalities
k^82^	1 Miller Fisher syndrome	50	M	anosmia; ageusia	
	1 polyneuritis cranialis	39	M		
Jebril^83^	cited: Desforges et al.^60^ and Filatov et el.^66^				
Moriguchi et al.^84^	1 meningitis/encephalitis	24	M		CT: no evidence of brain edema; 15 days later DWI: hyperintensity along the wall of inferior horn of right lateral ventricle; FLAIR: hyperintense signal changes in the right mesial temporal lobe and hippocampus with slight hippocampal atrophy; contrast-enhanced imaging showed no definite dural enhancement
Poyiadji et al.^68^	1 acute hemorrhagic necrotizing encephalopathy	>45	F		noncontrast CT: symmetric hypoattenuation within the bilateral medial thalami with a normal CT angiogram and CT venogram; MRI: hemorrhagic rim enhancing lesions within the bilateral thalami, medial temporal lobes, and subinsular regions
Sharifi-Razavi et al.^85^	1 intracerebral hemorrhage	79	M		CT: massive intracerebral hemorrhage in right hemisphere accompanied by intraventricular and subarachnoid hemorrhage
Zayet et al.^54^	1 acute encephalopathy				
Zhao et al.^86^	1 Guillain-Barré syndrome	61	F		
Zhou et al.^67^	1 viral encephalitis	56			

a CT, computed tomographic images; DWI, diffusion weighted images; F, female; FLAIR, fluid-attenuated inversion recovery images; M, male; MRI, magnetic resonance imaging.

The identified patient had anosmia and ageusia, but neither the presence of nasal obstructions nor the imaging findings was specified. These data are found in two other studies,^43,87^ each reporting the case of a patient with anosmia without nasal obstruction and no severe neurological complications. The imaging results of the two studies were conflicting because nasal congestion was not evident in one with magnetic resonance imaging (MRI),^87^ while in the other with both computed tomographic (CT) imaging and MRI the bilateral obstruction of the olfactory cleft was evident without obstruction of the rest of the nasal cavities.^43^ In both works, there were no volumetric alterations of the olfactory bulb.

## What is New after Another 8 Months of Study?

The bibliographic research conducted for the articles published from April 21 to November 24, 2020 allowed the identification of 600 papers. Of these, only 494 were truly related to the correlation between COVID-19 and chemosensory symptoms and were written in English.

**Figure 1:**
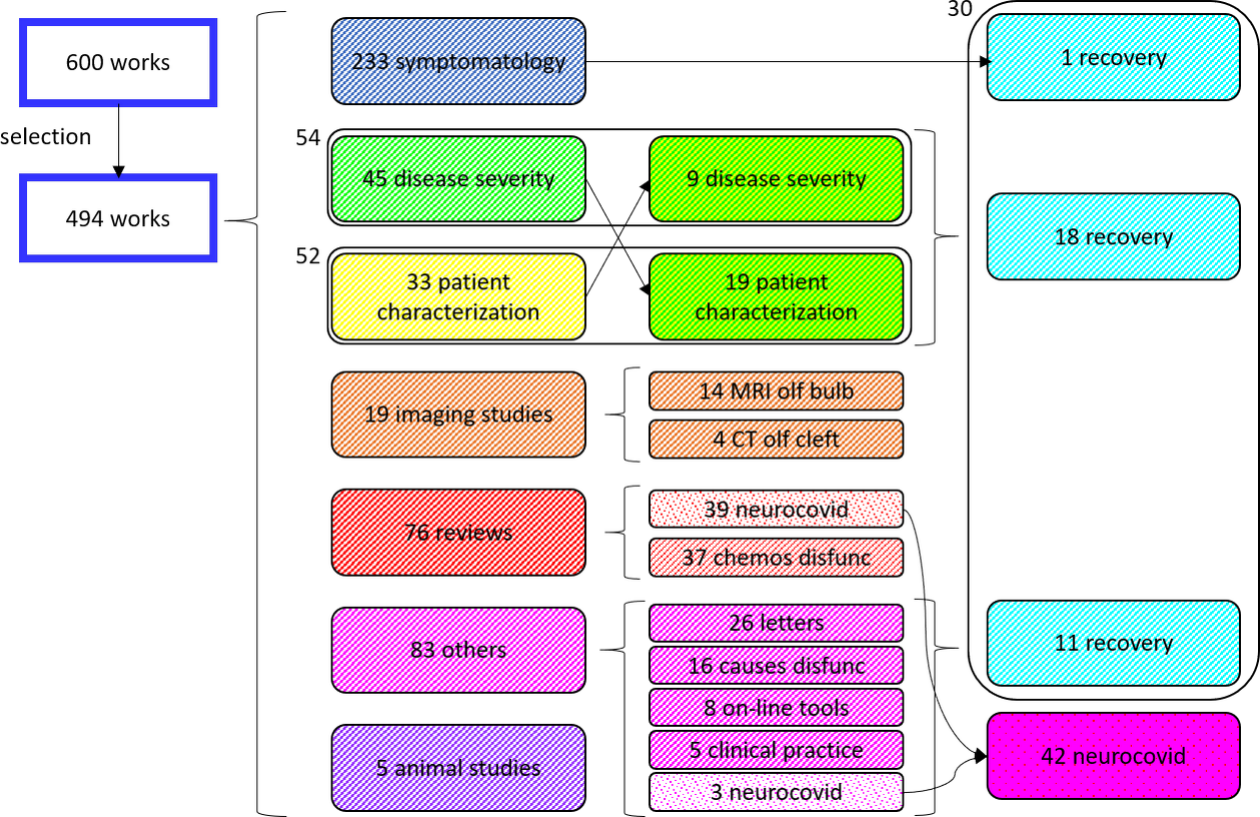
Categorization of published articles is not perfectly defined, as many papers deal with different aspects of chemosensory symptomatology. This is the case of the articles that try to identify the correlation between the symptoms and the severity of the disease and of those that try to define which subjects are most exposed. Works belonging to different categories also deal with the recovery of symptoms and the possible neurological sequelae of COVID-19. chemos, chemosensory; CT, computed tomographic image; disfunc, disfunctions; MRI, magnetic resonance image; olf, olfactory.

Figure 1 shows the division by type of the identified articles. The majority of articles (47.17%) reported chemosensory symptoms as one of the symptoms of COVID-19. Many reviews (48.68% of reviews) also referred to this aspect, while the others (51.32%) described the aspects that lead to considering COVID-19 a neurological disease. Imaging studies were aimed at demonstrating the spread of the virus in the CNS. Only four studies involved CT of the olfactory cleft, and the results were mixed. Fourteen documents referred to MRI of the olfactory bulb, and of these 71.43% identified anomalies.

An attempt was made to differentiate articles concerning the type of patients presenting with symptoms from those relating to disease severity. But in these papers the data often crossed, and it was found that 10.93% of the papers contained information on the severity of the disease and 10.53% included information on the type of patients suffering from chemosensory dysfunction.

Regarding the correlation between chemosensory symptoms and COVID-19 severity, 28 studies were conducted in homogeneous populations (23 on mild to moderate patients and 5 on hospitalized patients, i.e., patients with worse disease manifestation); therefore, it was not possible to perform the analysis. Two articles indicated an absence of correlation between symptomatology and severity, and 1 article indicated a correlation with asymptomatic patients, 28 with paucisymptomatic patients, and 6 with more severe patients.

Among the types of patients who most frequently presented chemosensory symptoms, 21.79% of the studies reported a correlation for patients with comorbidities (8/17 headache), 23.08% tried to identify a link with age, and 25.64% reported a possible link with gender. Sixteen out of 18 articles reported a correlation with age, identifying younger people as the subjects most affected by these symptoms. Seventeen of 20 articles reported a correlation with gender, with a prevalence of females over males (12 vs 5 manuscripts). And 6.07% of the studies are concerned with the recovery of chemosensory functionality. Three out of 30 articles reported that hyposmia recovered more rapidly than anosmia and both, however with longer times than general and sinonasal symptoms. Persistence of symptoms was found in five papers even when the SARS-CoV-2 swab gave negative results. Opinions were the most conflicting on recovery times and ranged from less than 1 month to more than 2 months. Only three papers specified that recovery times referred to recovery for most of the tested subjects. Some people recovered completely within a few weeks, while for others the symptoms lasted over time. Data on the absence of recovery seem absent. On the other hand, most of the tests for the evaluation of functionality were carried out on the basis of questionnaires administered at a distance and not of real objective tests. Four studies warned that the results obtained from objective tests were worse than those obtained from subjective, opinion-based tests.

## Discussion and Conclusion

The first anecdotal data, which however underline the obvious interest in the disease, make it clear how the need to identify paucisymptomatic or asymptomatic patients was felt immediately. In fact, the necessity to try to contain the spread of the disease in every way, especially considering its aggression, was immediately understood. The first real report with data from patients presenting chemosensory symptomatology was that of Giacomelli et al.^42^ By analyzing this work, we can make extremely interesting considerations:

- Both the olfactory and gustatory symptoms were not always present, and we can speculate that this depends on the different access route of the virus in the host.
- The people who presented this symptomatology seemed to be those for whom the effects of the infection to date are less serious and lethal, that is, females and young people.

These same considerations have also been the leitmotif of most of the scientific research conducted over the months to understand the type and degree of correlation between chemosensory symptoms and COVID-19 and to predict the possible future effects of the disease on the subjects involved.

Regarding the first single or few cases reports, the data confirmed the observations of Giacomelli et al.’s work.^42^ The data of the studies carried out on larger populations confirmed that chemosensory symptoms seem to be present with higher incidence in the milder forms of COVID-19. However, these data must be weighed considering the groups of patients studied.

The limits of the observations made are, in fact:

- The studies were aimed at understanding if chemosensory symptoms could identify asymptomatic and paucisymptomatic patients and, therefore, predominantly concerned patients with mild disease.
- The studies were carried out on patients, generally not hospitalized, to avoid bothering more compromised people and particularly in the case of patients recruited for the compilation of online questionnaires, only based on their answers.
- It is difficult to test the chemosensory symptomatology, especially without objective tests, but only on the basis of the responses of patients; these people are not always able to pay attention to these types of symptoms, especially at the onset of manifestations when dysfunctions and losses are minor.

This concept is also confirmed in Soler et al.^55^ First, patients rarely pay attention to these symptoms, and, for example, there is evidence of 3-year delays between the appearance of olfactory symptoms and the first tests.^19^ Second, it is difficult to carry out tests on infected patients.

It should be noted that it is not new for ENT specialists to see the senses of taste and smell altered by a virus. Olfactory and taste disorders are related with a wide range of viral infections.^18^ Viruses can cause an inflammatory reaction of the nasal mucosa resulting in the development of rhinorrhea.^20^ And it is also known that viruses can enter the CNS, in several ways, including the olfactory route. Indeed, the literature abounds with both animal studies^88−90^ and human studies.^63,91−93^ To be precise, the olfactory route can be the access road to the CNS not only for viruses but also for other environmental agents, such as prions and toxins.^94^

The works shown in Table 3 summarize the various possible access routes to the CNS by SARS-CoV-2. It is difficult to prove which path was really used by this virus, in the presence of brain damage certainly induced by the virus itself. The same correlation between brain damage and the virus is difficult to prove because it should be stated with certainty that the damage found was not pre-existing to the infection. Furthermore, it has been shown that the volume of the olfactory bulb decreases in postinfectious anosmia, without this leading to CNS involvement.^95^ In any case, the exact route by which SARS-CoV and MERS-CoV enter the CNS is still not known. Ultrastructural studies and/or studies to detect virus particles or viral antigens are needed to identify the access route of SARS-CoV-2.

The studies summarized in Table 3 have the particularity of completely changing the perspective in the evaluation of COVID-19. In the initial phase of the pandemic, COVID-19 was seen as an infection that manifested itself mainly with interstitial pneumonia. The neurotrophic potential and the possibility of a neuroinvasion of the CNS have led to a reconsideration of the entire symptomatic picture. Brain damage could be the main trigger in the severity of COVID-19, causing or exacerbating symptoms. For example, respiratory distress may be due in part to damage caused by the virus in the nuclei of the brain that control respiratory activity.^65^ Similar behavior by a virus, with similar sequelae, has been described for other viruses.^59^

Specifically, other viruses of the coronavirus family have shown similar behavior with access to the host, with invasion of the CNS with functional alterations in both the central and peripheral organs, and with consequent also serious secondary diseases. Many of these secondary pathologies are not inherent in the respiratory tract. For example, staying in the context of viruses belonging to the coronavirus family, in an animal study, it was shown that hemagglutinating encephalomyelitis virus (HEV) 67N strain, the first coronavirus, primarily infects suckling piglets through the oronasal pathway, and then is retrograde delivered through the peripheral nerves to the medullary neurons responsible for peristaltic function of the digestive tract, causing so-called vomiting diseases.^30^ The human coronavirus has been identified as a possible etiological agent for diseases outside the respiratory tract such as myocarditis, meningitis, severe diarrhea (and other gastrointestinal problems), and multiorgan failure.^60^ Evidence has also been reported for SARS,^96^ for MERS,^97^ and in general for coronavirus affecting children.^98^

The neurotropism and neuroinvasiveness that seem to characterize this virus combined with the neurological symptoms already evident in patients with COVID-19 led us to seriously consider the fact that this pathology is, first, a neural pathology. The first data on the lesions actually found in the brains of COVID-19 patients (Table 4) confirmed this hypothesis. However, this evidence is not surprising, given that already for SARS-CoV the presence of the virus had been detected in autopsy brain samples through immunohistochemical analysis, electron microscopy, and real-time reverse transcriptase-polymerase chain reaction (RT-PCR).^96^

Studies conducted in the past 8 months confirm all these considerations but do not clarify all aspects. Many studies, although excellent, have been conducted evaluating chemosensory symptoms using web-based questionnaires,^99−101^ with the already discussed problems/limitations that this method entails. Consequently, despite the large number of published articles, many questions still remain open. Which patients are most susceptible to chemosensory symptoms? The data so far seem to confirm that these are women and young people, that is, categories that seem to be affected by the less severe forms of the disease.^102−104^ What is the mechanism by which SARS-CoV-2 induces anosmia in these patients? In this work, 18 studies were identified that sought to explain the causes of this symptomatology and, in particular, the role of inflammation in anosmia. When COVID-19 infection occurs in the upper or lower respiratory tract, it may cause a wide spectrum of symptoms, from mild to highly acute respiratory syndrome, and trigger the release of proinflammatory cytokines, including interleukin IL-1β and TNF-α. When COVID-19 binds to the toll-like receptor (TLR), pro-IL-1β is released. Furthermore, pro-IL-1β is cleaved by captase-1, which leads to inflammasome activation and production of active mature IL-1β. Then, active mature IL-1β can mediate fever, lung inflammation, and fibrosis. Interestingly, it has been proved that when proinflammatory IL-1 family members are suppressed, they might have therapeutic effects on many inflammatory diseases (such as viral infections).^105,106^ Another still unanswered question is how are chemosensory symptoms related to COVID-19 severity? The symptomatology is present both in asymptomatic affected patients, both in patients with mild-to-moderate forms and in patients with severe forms, although it seems that there is a greater involvement for the less severe forms.^102,103,107,108^ The recovery of these symptoms is still undetermined; i.e., it is not yet known whether it is total or partial and what the recovery times are.^103,109,110^ And this question refers to what is perhaps the most important question: will there be neurological sequelae in the medium-to-long-term? Incomplete resolution of chemosensory alterations could indicate future neurodegenerative diseases, as these symptoms are prognostic symptoms of many neurodegenerative diseases. The limited imaging data currently available does not allow for speculation. Although studied in every corner of the world, many of the peculiarities of SARS-CoV-2 and its manifestation in COVID-19 still remain unknown and/or to be understood.

## Methods

A systematic literature review of the PubMed and Google Scholar databases was performed of all studies published up to April 20, 2020 to identify all relevant articles. We considered 1 month of literature starting from March 20, 2020, the date of the first formal position of an ENT scientific organization regarding chemosensory symptoms. Combined search included the terms: “COVID-19 or SARS-CoV-2 and olfactory disfunction, olfactory disorder, smell disfunction, smell disorder, hyposmia, anosmia”. We included only articles that were in English. Studies were excluded if they did not have an associated and accessible full paper.

Further research was conducted on the basis of the studies identified through a careful analysis of their references. The articles found in this way were selected utilizing the same criteria used in the previous search.

Subsequently, a systematic review of the PubMed database of all studies published between April 21 and November 24, 2020 was performed utilizing the same criteria, already used for the first search.

## Abbreviations

AAO-HNS: American Academy of Otolaryngology – Head and Neck Surgery
ACE2: Angiotensine Converting Enzyme 2
CNS: Central Nervous System
COVID-19: Coronavirus disease 19
CT: Computed Tomographic image
HEV: Hemagglutinating Encephalomyelitis Virus
MERS-CoV: Middle East Respiratory Syndrome-related Coronavirus
MRI: Magnetic Resonance Image
RT-PCR: Real-time reverse Transcriptase-Polymerase Chain Reaction
SARS-CoV: Severe Acute Respiratory Syndrome-related Coronavirus
SARS-CoV-2: Severe Acute Respiratory Syndrome-related Coronavirus 2
WHO: World Health Organization
